# Methyl phenlactonoates are efficient strigolactone analogs with simple structure

**DOI:** 10.1093/jxb/erx438

**Published:** 2017-12-28

**Authors:** Muhammad Jamil, Boubacar A Kountche, Imran Haider, Xiujie Guo, Valentine O Ntui, Kun-Peng Jia, Shawkat Ali, Umar S Hameed, Hidemitsu Nakamura, Ying Lyu, Kai Jiang, Kei Hirabayashi, Masaru Tanokura, Stefan T Arold, Tadao Asami, Salim Al-Babili

**Affiliations:** 1 King Abdullah University of Science and Technology, Division of Biological and Environmental Sciences and Engineering, Thuwal, Saudi Arabia; 2 King Abdullah University of Science and Technology, Computational Bioscience Research Center, Division of Biological and Environmental Sciences and Engineering, Thuwal, Saudi Arabia; 3 Graduate School of Agricultural and Life Sciences, University of Tokyo, Tokyo, Japan

**Keywords:** Carlactone, methyl carlactonoic acid, plant architecture, root parasitic weeds, *Striga hermonthica*, strigolactones, tillering

## Abstract

Strigolactones (SLs) are a new class of phytohormones that also act as germination stimulants for root parasitic plants, such as *Striga* spp., and as branching factors for symbiotic arbuscular mycorrhizal fungi. Sources for natural SLs are very limited. Hence, efficient and simple SL analogs are needed for elucidating SL-related biological processes as well as for agricultural applications. Based on the structure of the non-canonical SL methyl carlactonoate, we developed a new, easy to synthesize series of analogs, termed methyl phenlactonoates (MPs), evaluated their efficacy in exerting different SL functions, and determined their affinity for SL receptors from rice and *Striga hermonthica*. Most of the MPs showed considerable activity in regulating plant architecture, triggering leaf senescence, and inducing parasitic seed germination. Moreover, some MPs outperformed GR24, a widely used SL analog with a complex structure, in exerting particular SL functions, such as modulating Arabidopsis roots architecture and inhibiting rice tillering. Thus, MPs will help in elucidating the functions of SLs and are promising candidates for agricultural applications. Moreover, MPs demonstrate that slight structural modifications clearly impact the efficiency in exerting particular SL functions, indicating that structural diversity of natural SLs may mirror a functional specificity.

## Teaser

We developed easy to synthesize and efficient strigolactone analogs with great application potential. Biological activities and receptor binding assays demonstrate the effect of structural modification on the efficacy and specificity of strigolactones.

## Introduction

Strigolactones (SLs) are secondary metabolites originally identified as root-derived chemical signals inducing seed germination in root parasitic plants of the family Orobanchaceae (Xie *et al.*, 2010). Later, SLs were also shown to induce hyphal branching in arbuscular mycorrhizal (AM) fungi, which is required for establishing beneficial AM symbiosis (Akiyama *et al.*, 2005). In the meantime, SLs were recognized as a novel class of plant hormones that determine different developmental processes, such as establishing shoot and root architecture, regulating secondary growth and inducing senescence (Gomez-Roldan *et al.*, 2008; Umehara *et al.*, 2008; Ruyter-Spira *et al.*, 2013; Beveridge, 2014). In addition, SLs are involved in pathogen defense and act as positive regulators of abiotic stress responses (Ha *et al.*, 2014; Torres-Vera *et al.*, 2014; Decker *et al.*, 2017).

Natural SLs are carotenoid derivatives consisting of a lactone ring (D-ring) that is connected by an enol ether bridge (in *R*-configuration) to a structurally variable second moiety (Al-Babili and Bouwmeester, 2015). Canonical SLs, such as strigol and orobanchol, contain a tricyclic lactone (ABC-ring) as a second moiety and are divided, depending on stereochemistry of the B/C-junction, into strigol-like (β-orientation, up) and orobanchol-like (α-orientation, down) SLs (Ueno *et al.*, 2011; Xie *et al.*, 2013). Besides the stereochemistry of the B/C-junction, various modifications of the ABC-ring, such as hydroxylation at different positions, lead to the diversity of canonical SLs. Non-canonical SLs, such as methyl carlactonoate (Abe *et al.*, 2014), heliolactone (Ueno *et al.*, 2014) and zealactone (Charnikhova *et al.*, 2017; Xie *et al.*, 2017), do not have an ABC-lactone as a second moiety. The question of whether and how structural diversity leads to functional specificity of the around 25 known natural SLs is largely elusive (Xie, 2016).

The availability of SL biosynthesis and perception mutants has enabled the elucidation of key steps in SL biosynthesis and perception (Al-Babili and Bouwmeester, 2015; Lumba *et al.*, 2017; Waters *et al.*, 2017). SL biosynthesis starts in plastids with the reversible *cis*/*trans*-isomerization of the precursor all-*trans*-β-carotene into 9-*cis*-β-carotene (Alder *et al.*, 2012; Bruno and Al-Babili, 2016). In the next step, the stereospecific carotenoid cleavage dioxygenase 7 (CCD7) cleaves 9-*cis*-β-carotene into the intermediate 9-*cis*-β-apo-10′-carotenal and β-ionone (Alder *et al.*, 2012; Bruno *et al.*, 2014). Another CCD, CCD8, converts 9-*cis*-β-apo-10′-carotenal via a combination of repeated dioxygenation and intra-molecular rearrangements into carlactone (Alder *et al.*, 2012; Bruno *et al.*, 2017). Carlactone is a central metabolite of SL biosynthesis (Alder *et al.*, 2012; Seto *et al.*, 2014) and is the substrate for cytochrome P450 enzymes of the clade 711, MAX1, in Arabidopsis (Booker *et al.*, 2005), which catalyse the formation of canonical SLs, such as 4-deoxyorobanchol, and non-canonical SLs, such as carlactonoic acid (Abe *et al.*, 2014; Zhang *et al.*, 2014). In Arabidopsis carlactonoic acid is methylated by an unidentified methyltransferase into methyl carlactonoate (Abe *et al.*, 2014). In the next step, methyl carlactonoate is hydroxylated by LATERAL BRANCHING OXIDOREDUCTASE (LBO) into an unidentified product that may be the final product in Arabidopsis SL biosynthesis (Brewer *et al.*, 2016). The rice MAX1, homolog carlactone oxidase, catalyses the conversion of carlactone into 4-deoxyorobanchol (*ent*-2′-*epi*-5-deoxystrigol), the precursor of canonical, orobanchol-like SLs. Orobanchol itself is produced by another rice MAX1 homolog, the orobanchol synthase (Zhang *et al.*, 2014; Al-Babili and Bouwmeester, 2015).

Strigolactone perception and downstream signaling involve the α/β-fold hydrolase DWARF14 (D14) (Hamiaux *et al.*, 2012; de Saint Germain *et al.*, 2016; Yao *et al.*, 2016), which acts as a non-canonical receptor that covalently binds the D-ring of SLs during their hydrolysis. In addition, SL signaling requires the leucine-rich-repeat F-box protein MORE AXILLARY GROWTH 2 (MAX2)/DWARF3 (D3) (Stirnberg *et al.*, 2007), a subunit of a SKP1-CUL1-F-box-protein (SCF)-type ubiquitin ligase complex, which targets repressors of SL signaling, such as Arabidopsis SUPPRESSOR OF MORE AXILLARY GROWTH2 1-LIKE 6,7,8 (SMXL6,7,8) or rice DWARF53 (D53), for proteasome-mediated degradation (Jiang *et al.*, 2013; Zhou *et al.*, 2013; Soundappan *et al.*, 2015; Wang *et al.*, 2015; Liang *et al.*, 2016). The F-box protein MAX2 is also required for signal transduction of karrikins, smoke-derived compounds that likely mimic an unidentified internal signaling molecule(s) and that inhibit hypocotyl growth and induce seed germination in various plant species but not in root parasitic weeds (Nelson *et al.*, 2012). Karrikins share structural similarities (D-ring) with SLs and bind to the D14 paralog KARRIKIN INSENSITIVE 2 (KAI2), likely leading to proteasomal degradation of presumed suppressors (Waters *et al.*, 2017). Karrikin response is also triggered by the 2′*S*-configured stereoisomer of the common SL analog GR24 that is usually applied as a racemic mixture of 2′*S* and 2′*R* isomers (Scaffidi *et al.*, 2014). The genome of the root parasitic plant *Striga hermonthic*a encodes 11 D14/KAI2 homologs. It was recently shown that *Striga* KAI2 paralogs, especially ShHTL7, which constitute a distinct clade, are responsible for perception of host-released SLs and thus for triggering parasitic weed seed germination (Conn *et al.*, 2015; Tsuchiya *et al.*, 2015; Yao *et al.*, 2017).

Several parasitic *Striga* and *Phelipanche* species of the family Orobanchaceae are of great importance for agriculture. The *Striga* species *S. asiatica* and *S. hermonthica* infect cereals, including maize, sorghum, pearl millet, and rice, while *Phelipanche* species affect crops such as sunflower, tomato and legumes (Parker, 2009). *S. hermonthica* is considered as one of the seven most severe biotic threats to food security, affecting subsistence and livelihood of 100 million people in sub-Saharan Africa (Pennisi, 2010). *S. hermonthica* has been observed in 32 countries (Rodenburg *et al.*, 2017) infesting an estimated 50 million hectares of arable land in the Sahel and savanna zones in Africa, causing annual losses of around 7 billion US$ (Ejeta, 2007; Parker, 2009, 2012). Related broom rape species, such as *Orobanche crenata* and *Phelipanche ramosa*, parasitize several non-cereal crops causing severe yield losses in the Mediterranean, central and eastern Europe, and Asia (Parker, 2009). Heavy soil infestation with enormous numbers of long-lived, tiny seeds, and germination dependency of these seeds on host-derived signaling molecules has made control of these parasitic weeds very difficult (Joel, 2000; Ejeta, 2007; Delavault *et al.*, 2017).

During the evolution of parasitism, root parasitic plants have either totally lost or significantly reduced their capability for photosynthesis and, hence, cannot survive without a host plant that provides them with metabolites, water and minerals (Xie *et al.*, 2010). In this parasite–host relationship, SLs are chemical signals required for the germination of parasite seeds, ensuring availability of an appropriate host (Ruyter-Spira *et al.*, 2013). Such dependency on germination cues may provide opportunities for parasitic weed control. Induction of parasitic weed seed germination by exogenous application of germination stimuli before sowing crop seeds could, for instance, lead to the death of emerging parasite seedling (suicidal germination). This could be a promising approach to reduce the weed seed bank in soil. For this purpose, natural SLs are, however, not suitable candidates since they cannot be obtained from natural sources in sufficient quantities or at reasonable costs (Zwanenburg *et al.*, 2016*b*). Moreover, organic synthesis of natural SLs is challenging, due to their complex structures that contain several chiral centers (Kgosi *et al.*, 2012; Zwanenburg and Pospísil, 2013; Zwanenburg *et al.*, 2016*a*). Hence, a prerequisite for implementing the suicidal germination approach outlined above is the availability of SLs or analogs/mimics that are easy to synthesize and that efficiently induce *Striga* seed germination (Oancea *et al.*, 2017; Dvorakova *et al.*, 2017). SL analogs could also be used in agriculture and horticulture, for instance, to direct water and other resources in one major branch by suppressing branching or tillering, to induce secondary growth, to enhance abiotic stress tolerance or to modulate root architecture (Li *et al.*, 2016), by increasing primary root length (Agusti *et al.*, 2011; Ha *et al.*, 2014). SLs also accelerate senescence (Yamada *et al.*, 2014), a functionality that might be exploited for the development of a new suite of herbicides.

Recently, we have reported on a carlactone-based SL analog, nitro-phenlactone, which has SL activity, but with different efficiency, indicating the possibility of establishing SLs analogs with specific functions (Jia *et al.*, 2016). In the present work, we developed a new series of SLs analogs, methyl phenlactonoates (MPs), which can be easily synthesized and which resemble the non-canonical SL methyl carlactonoate. Aiming at the identification of analogs that can be applied as suicidal germination agents and growth regulators or used in basic research to determine particular SL activities, we measured the stability of MPs, explored their activities in exerting different SL functions and investigated their affinity for SL receptors.

## Materials and methods

### Chemical synthesis of methyl phenlactonoates

**Fig. 1.**
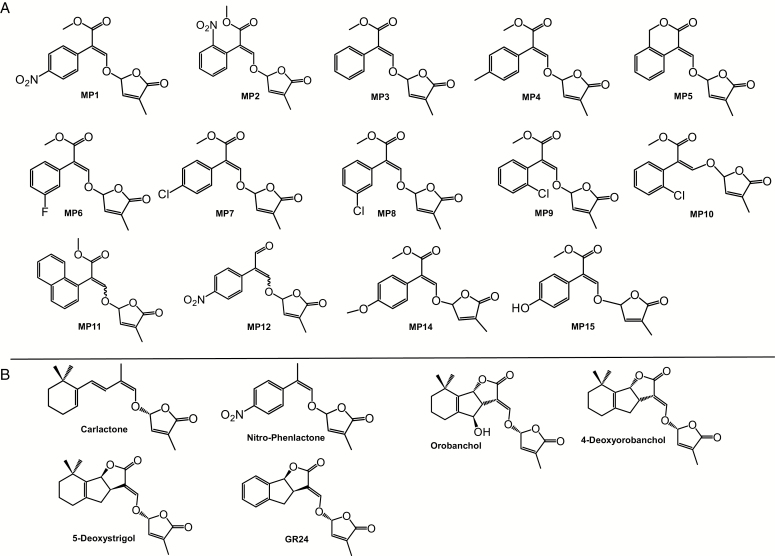
(A) Structure of methyl phenlactonoates (MPs). (B) Structure of carlactone, nitro-phenlactone, orobanchol, 4-deoxyorobanchol, 5-deoxystrigol and the standard strigolactone analog, GR24.

**Fig. 2.**
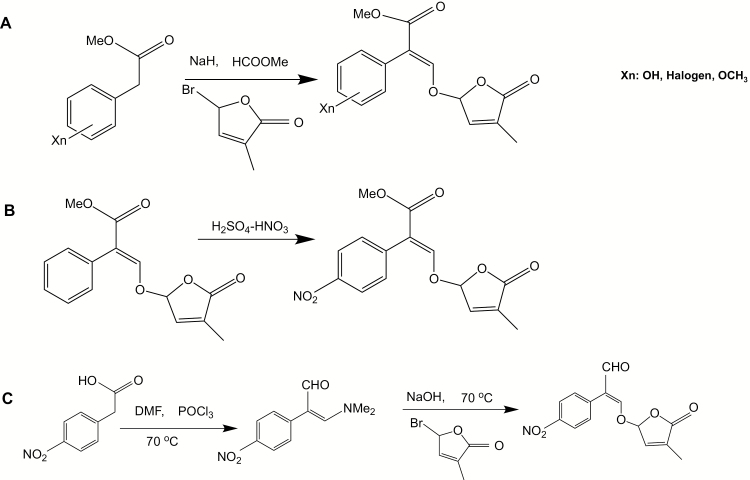
Synthesis of MPs. Most of the MPs, except MP1 and MP12, were synthesized by following the procedure as described previously (Mangnus *et al.*, 1992*a*). (A) Synthesis of MP3, (*E*)-methyl 3-(4-methyl-5-oxo-2,5-dihydrofuran-2-yloxy)-2-phenylacrylate. (B) Synthesis of MP1, (*E*)-methyl 3-(4-methyl-5-oxo-2,5-dihydrofuran-2-yloxy)-2-(4-nitrophenyl)acrylate. (C) Synthesis of MP12, (*E*)-3-(4-methyl-5-oxo-2,5-dihydrofuran-2-yloxy)-2-(4-nitrophenyl)acrylaldehyde.

All compounds (Fig. 1) synthesized here were produced and applied as a racemic mixture of two stereoisomers that differ in the configuration of the C2′ atom (2′*R* and 2′*S* configurations). For the synthesis of (*E*)-methyl 3-(4-methyl-5-oxo-2,5-dihydrofuran-2-yloxy)-2-phenylacrylate (MP3), we used a previously described protocol (Mangnus *et al.*, 1992*a*). Sodium hydride (372 mg, 3.32 mmol) was added to a cooled (0 °C) and stirred solution of methyl phenylacetate (2.0 g, 13.3 mmol) and methyl formate (1.1 ml, 18.0 mmol) in dry tetrahydrofuran (THF; 27 ml) in a 100 ml Erlenmeyer flask. After 10 min, the reaction mixture was warmed to room temperature and stirred overnight under elevated ambient nitrogen. Then the reaction mixture was cooled again with an ice bath, and 5-bromo-3-methyl-2(5*H*)-furanone (2.4 g, 13.3 mmol) in THF (5 ml) was gradually added. The mixture was stirred at room temperature for 2 h. The reaction mixture was poured into ethyl acetate (70 ml), and organic layer was washed successively with water (80 ml) and saturated sodium chloride solution (80 ml), dried with sodium sulfate, and concentrated *in vacuo*. Oily residue was purified with silica gel (Wakosil^®^ C-300HG) column with hexane and ethyl acetate as eluent to give title compound MP3 (Fig. 2A). Physico-chemical properties of MP3 are shown in Supplementary Table S1 at *JXB* online. The compounds MP2, MP4-11, MP14 and MP15 were synthesized following the same procedure by using accordingly substituted methyl phenylacetates as starting materials.

(*E*)-Methyl 3-(4-methyl-5-oxo-2,5-dihydrofuran-2-yloxy)-2-(4-nitrophenyl) acrylate (MP1) was prepared by nitration of MP3 (Fig. 2B). In a 10 ml round-bottom flask, 0.4 ml of concentrated sulfuric acid was added drop-wise to 1 mmol of MP3, and cooled with an ice bath. After complete addition of sulfuric acid, approximately 0.2 ml of concentrated nitric acid was added drop-wise with cooling by a small graduated plastic pipette and mixed by gentle swirling. The reaction mixture was then allowed to stand at room temperature for about 15 min and poured into 10 ml of ice water with stirring. Organic chemicals were then extracted three times with ethyl acetate. After evaporation *in vacuo*, the residue was purified using a silica gel column with ethyl acetate–hexane as eluents to give nitrated compounds (65% yield). These nitrated compounds, 2-nitro (MP2) and 4-nitro (MP1), were separated by a reverse-phase column (ODS) with 1:1 MeOH:water. Physico-chemical properties of MP1 are shown in Supplementary Table S1.

(*E*)-3-(4-Methyl-5-oxo-2,5-dihydrofuran-2-yloxy)-2-(4-nitrophenyl) acrylaldehyde (MP12) was synthesized as follows. To a stirred solution of 4-nitrophenyl acetic acid (1.8 g) in dimethylformamide (5 ml), POCl_3_ (2.9 ml, 30 mmol) was added slowly (over 15 min) so that the reaction temperature was kept below 70 °C. Then the reaction mixture was stirred at 70 °C for another 12 h. The mixture was poured into 10 g of ice and neutralized by K_2_CO_3_ solution (200 ml). The resultant solid was isolated by filtration and dried to give (*Z*)-3-(dimethylamino)-2-(4-nitrophenyl)acrylaldehyde, which was used in the next reaction without further purification. (*Z*)-3-(Dimethylamino)-2-(4-nitrophenyl)acrylaldehyde (150 mg, 0.68 mmol) and 7.7 M NaOH aq 97 μl (1.1 eq, 0.75 mmol) was heated at 70 °C until the reaction mixture became homogeneous. Then the reaction mixture was dried by evaporation under reduced pressure. To the solution of resultant residue in DMSO (1.5 ml), 5-bromo-3-methyl-2(5*H*)-furanone (120 mg, 0.68 mmol) was added slowly and stirred overnight. The reaction mixture was diluted with diethylether and the organic layer was washed successively with water and saturated sodium chloride solution, dried (Na_2_SO_4_) and evaporated (Fig. 2C). The residue was column chromatographed on silica gel using a mixture of hexane and ethyl acetate as eluent to give MP12. Physico-chemical properties of MP12 are shown in Supplementary Table S1.

### Chemical stability

Aqueous solutions of MPs (50 μg ml^−1^; pH 6.8) were incubated at 21 °C in amber HPLC vials (adapted from de Saint Germain *et al.*, 2016). For sample preparation, 50 μl of an acetone solution (1 mg ml^−1^) was diluted to the final concentration with methanol (425 μl), water (500 μl) and 25 μl of 1-indanol (1.0 mg ml^−1^ solution in acetone) as internal standard. The time course of degradation was monitored by UHPLC analysis on a Dionex Ultimate 3000, using a Zorbax Eclipse Plus C_18_ column (3.5 μm, 2.1 × 150 mm). The column was developed at a flow rate of 0.6 ml min^–1^ at 35 °C with a linear gradient from 5% to 95% acetonitrile in water within 15 min, maintaining the final conditions for another 4 min. The column was operated at 35 °C with a flow rate of 0.25 ml min^−1^. Compounds eluted from the column were detected with a diode array detector. The relative quantity of remaining (non-degraded) product was determined by comparison with the internal standard. Stability was monitored at 24 h intervals up to 3 weeks.

### Parasitic weed seed germination bioassays

Parasitic weed seeds’ (*S. hermonthica* and *P. ramosa*) germination activity was studied as described previously (Jamil *et al.*, 2012). Six pre-conditioned seed discs were placed in a 90 mm Petri dish containing a filter paper ring wetted with 0.9 ml sterile MilliQ water. Then 50 μl of SL analog solution (10^–5^–10^–12^ M) was applied on each of the six discs, for each concentration. GR24 solutions with equal concentrations and sterile MilliQ water were included as a positive and negative control, respectively. After application, seeds of *S. hermonthica* and *P. ramosa* were incubated in dark at 30 °C for 2 d and at 25 °C for 4 d, respectively. Germination (seeds with radicle emerging through the seed coat) was scored under a binocular microscope, and germination rate (%) was calculated.

### Rice micro tillering bioassays

Rice seeds (WT, *d3*, *d10*) were surface-sterilized by washing with 70% ethanol for 1 min and then with 2.5% sodium hypochlorite for 15 min. Seeds were then rinsed thoroughly with sterile MilliQ water and incubated in water for 2 d at 30 °C in the dark. Pre-germinated seeds were transferred to filter papers containing half-strength MS medium in 90 mm Petri dishes and incubated at 30 °C under fluorescent white light (130–180 μmol m^−2^ s^−1^) for 1 week. Seven-day-old seedlings were transferred to 50 ml falcon tubes (one seedling per tube) containing modified half-strength Hoagland nutrient solution and grown in greenhouse. Plants were treated with MPs at 2.5 μM to 2.5 × 10^–7^ μM, using GR24 as positive control. The compounds were applied six times, twice a week. Number of tillers per plant, plant height and fresh biomass were measured at final harvest.

### Measurement of dark-induced leaf senescence

One-week-old rice (*O. sativa*, var. Shiokari) seedlings were established as mentioned above. Uniform seedlings were transferred to 50 ml tubes containing half-strength Hoagland nutrient solution for 7 d and grown in an incubator under white fluorescent light (130–180 μmol m^−2^ s^−1^) with 16 h/8 h (L/D) at 28 °C for 1 week. Leaf segments of 2 cm were cut from middle part of third leaves of rice plants. Each segment was put in a well (in 24-well plates) containing 2 ml of 2.5 mM MES buffer with 0.05% Tween-20, and incubated at 30 °C in the dark for 7 d. After application of MPs, color change, chlorophyll content, ion leakage, and gene expression were monitored daily for 7 d.

### Arabidopsis hypocotyl elongation assays

Sterilized Arabidopsis seeds were sown on half-strength MS (with 0.5% sucrose+1% agar, 0.5 g l^−1^ MES, pH 5.7) plates supplemented with MPs or GR24 (at 1.0 μM). Plates were stored at 4 °C in darkness for 3 d. To initiate germination, plates were exposed to continuous white light for 24 h, then transferred to continuous monochromatic red light (20 μmol m^−2^ s^−1^, 22 °C) conditions for another 4 d. For hypocotyl length measurement, at least 30 seedlings were measured using the freely available ImageJ software (http://rsbweb.nih.gov/ij/) after taking digital photographs. A monochromatic red light source was applied as described previously (Wu and Yang, 2010). Light flow rates were measured using a Li250 quantum photometer (Li-Cor, Lincoln, NE, USA).

### Measurement of Arabidopsis lateral root density and primary root length

Sterilized Arabidopsis seeds were sown on half-strength MS (with 0.5% sucrose+1% agar, 0.5 g l^−1^ MES, pH 5.7) plates supplemented with GR24 or MPs (at 1.0 μM). Plates were stored at 4 °C in darkness for 3 d and then vertically grown at 22 °C in a Percival incubator under long day condition (16 h at 22 °C/8 h at 16 °C day/night, 60% relative humidity, 60–70 μmol m^−2^ s^−1^ white light) for 8 d. For determining the effect on lateral root density and primary root length, at least 30 seedlings were measured using ImageJ software after scanning roots.

### Protein expression and purification

*Striga hermonthica* ShHTL7 cDNA was used, GenBank accession KR013127, kindly provided by Tsuyoshi Ota, The University of Tokyo, Japan. Arabidopsis D14 (*AtD14*) cDNA, GenBank accession AY097402, was synthesized and cloned into pUC57 (GenScript). ShHTL7 and OsD14 cDNAs were amplified by RT-PCR using the primers shown in Supplementary Table S2, digested with *Bam*HI and *Xho*I, and ligated into *Bam*HI/*Xho*I-digested pGEX-6P-1 expression vector (GE Healthcare). Integrity of plasmids was confirmed by sequencing (KAUST Bioscience Core Lab). The plasmids were then transformed into *E. coli* BL21 (DE3) cells. The cells were grown in LB broth containing ampicillin (100 mg ml^−1^), incubated at 37 °C until OD_600_ of 0.6 and induced with 0.1 mM isopropyl-β-D-thiogalactopyranoside at 16 °C for 16 h. Harvested cells were resuspended in lysis buffer: 50 mM Tris–HCl (pH 8.0), 200 mM NaCl, 0.5% Tween-20 and 2 mM dithiothreitol (DTT). After sonication on ice for 10 min, the lysate was centrifuged at 75 000 *g* for 30 min at 4 °C. Supernatant was allowed to bind to glutathione–sepharose beads (GE Healthcare) for 2 h at 4 °C, washed 3 times with buffer (50 mM Tris-HCl (pH 8.0), 200 mM NaCl, 2 mM DTT), and eluted by cleaving the GST moiety with PreScission™ Protease (GE Healthcare) at 4 °C overnight. Eluted protein was further purified by gel filtration using a HiLoad 16/60 Superdex200 prep grade column (GE Healthcare) on an AKTA prime system (GE Healthcare) in gel filtration buffer (10 mM HEPES pH 7.5, 50 mM KCl and 2 mM DTT). Protein eluted as a sharp single peak and purity was judged by 4–15% SDS-PAGE (Bio-Rad). Purified protein was concentrated using an Amicon 10K filter unit (Merck Millipore) and stored at −80 °C until use. A similar expression and purification protocol was followed for OsD14 protein, except that OsD14 was eluted from glutathione resins using 0.2 mM reduced glutathione (Sigma-Aldrich) without cutting the GST tag.

### Intrinsic tryptophan fluorescence assays

ShHTL7/D14 tryptophans were excited at 280 nm and emission intensity was measured at 333 nm. ShHTL7/D14 (10 μM) was incubated at various dilutions (0.2, 0.4, 0.8, 1.6, 3.12, 6.25, 12.5, 25, and 50 μM) of GR24 and MPs for 30 min at room temperature before measurement. Each concentration point was measured in triplicate. Emitted fluorescence was monitored using a spectraMaxi3 plate reader (Molecular Devices) in 96-well black plates. Changes in tryptophan fluorescence intensity occur due to conformational changes in protein when it is bound to ligand; differences in fluorescence intensity were recorded and analysed. Data were normalized and dissociation coefficient (*K*_d_) values were calculated by fitting to a binding saturation single-site model with Prism 6 (GraphPad).

### *In vitro* hydrolysis assays

The hydrolysis of GR24 and MPs by ShHTL7/OsD14 was performed in a total volume of 0.5 ml of phosphate-buffered saline containing 10 µM of substrate. Purified ShHTL7/OsD14 was added at a concentration of 50 µg ml^−1^ and incubated for the indicated time at 37 °C. After adding 1-indanol (10 µl of a 2.5 mg ml^−1^ solution in methanol), as internal standard, solutions were filtered and transferred to HPLC vials. Hydrolysis of substrates was monitored by HPLC analysis using a Zorbax Eclipse Plus C_18_ column (3.5 µm, 2.1 × 150 mm), eluted by a gradient from 10% to 90% acetonitrile in water within 15 min, keeping the final condition for 4 min. The column was operated at 30 °C with a flow rate of 0.2 ml min^−1^. Eluted compounds were detected using a diode array detector. Amounts of remaining substrates were determined by calculating corresponding peaks, in comparison with an internal standard.

### Statistical analyses

Statistical analyses were carried out using the statistical software package R (version 3.2.2). Dose–response curves and half-maximum effective concentrations (EC_50_) were calculated to determine optimum amount of MPs. The synthetic strigolactone analog GR24 was used as reference to estimate efficacy of selected compounds. EC_50_ and estimated response were calculated using the drc package (https://cran.r-project.org/web/packages/drc/) with a four parameter logistic curve (dose–response curve) (Ritz and Streibig, 2005).

## Results

### Synthesis and chemical stability of MPs

We synthesized 14 methyl phenlactonoates (MPs) as described above in ‘Material and methods’ (structures are shown in Fig. 1 and synthesis steps in Fig. 2) and determined their stability, which is a decisive factor for their activity and application. Stability measurements were performed in comparison with GR24, using aqueous solution (pH 6.8) of the different compounds. MP2, MP3, MP4, MP5, MP7, and MP10 were more stable than GR24, with MP5 being the most stable compound (see Supplementary Fig. S1). MP12, which carries an aldehyde instead of carboxy methyl group, was quite unstable and not detectable even after only 24 h. MP1 and MP15 were slightly less stable than GR24, while the stability of MP6, MP8, MP9, MP11, and MP14 was very similar to that of GR24. Varying substitutions on different sites in the A-ring (phenol ring) in SLs could lead to variations in MP stability that finally might define the effectiveness of MPs as SLs.

### MPs are potent inducers of seed germination in root parasitic weeds

**Fig. 3.**
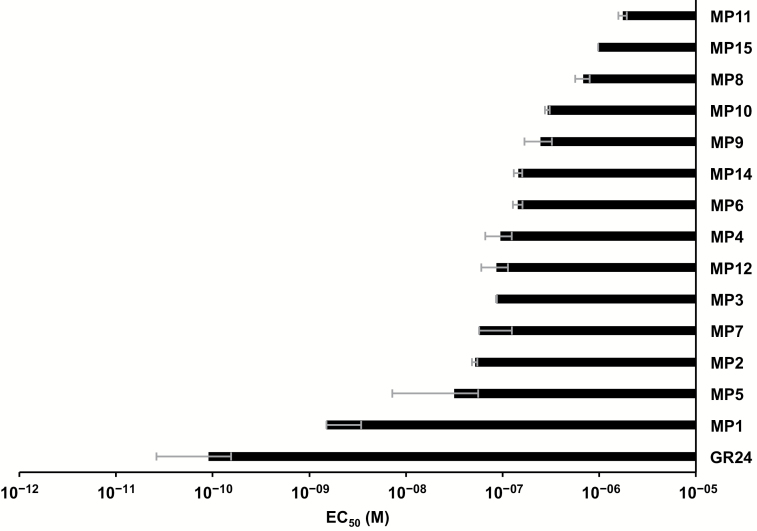
EC_50_ (half-maximal effective concentration) of methyl phenlactonoates for *Striga* seed germination. Concentrations ranging from 10^–5^ to 10^–12^ M were applied in a 50 μl volume on a disc containing 50–100 pre-conditioned *Striga* seeds. GR24 is included as positive control.

We determined the activity of MPs in inducing seed germination of the root parasitic weeds *S. hermonthica* and *P. ramosa*. In the first assay, we applied MPs at a 1.0 μM concentration. All compounds showed considerable activity in the *S. hermonthica* germination assay (see Supplementary Fig. S2) with rates ranging from 32% (MP11) to 75% (MP1). Differences between MPs were more pronounced in the *P. ramosa* germination assay where we observed much higher activity with MP1, MP2, and MP8, compared with other MPs (Supplementary Fig. S2). Next, we established half-maximal effective concentration (EC_50_) for *S. hermonthica* seed germination, using concentrations ranging from 10^–5^ to 10^–12^ M. Among MPs, we observed the highest activity with MP1, which exhibited an EC_50_ value of 1.5 × 10^–9^ M. Other MPs were at least ~5-fold weaker, showing EC_50_ values ranging from 3.2 × 10^–8^ (MP2) to 1.7 × 10^–6^ M (MP11). The EC_50_ of the lead compound, MP3, was 8.7 × 10^–8^ M. GR24 was the most active analog, exhibiting an EC_50_ value of 9.1 × 10^–11^ M (Fig. 3).

### MPs rescue the tillering phenotype in rice *d10* mutant

**Fig. 4.**
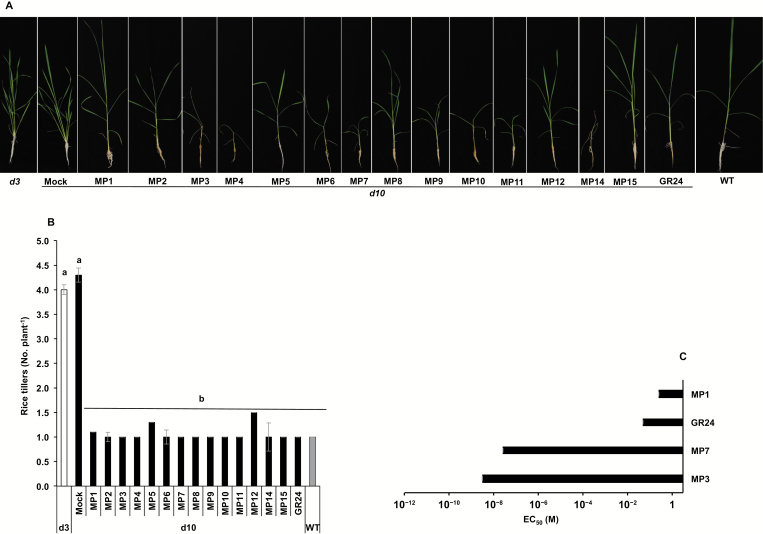
Rice tillering inhibition by methyl phenlactonoates. (A) Tillering phenotype of *d10* mutant in response to MPs. Methyl phenlactonoates were applied (2.5 μM) to 1-week-old rice seedlings (Shiokari, *d3*, *d10*) grown hydroponically in 50 ml tube twice a week up to 3 weeks. MP3 treatment led to growth retardation and senescence. (B) Number of tillers per plant counted after 3 weeks of MP application. Data are means±SE (*n*=8). Means not sharing a letter in common differ significantly at *P*_0.05_. (C) EC_50_ (half-maximal effective concentration) of selected MPs for tillering inhibition of the rice *d10* mutant.

Next, we tested whether MPs restore wild type tillering in the rice *d10* mutant. For this purpose, we grew the plants in hydroponic culture and applied the compounds at a 2.5 μM concentration. As shown in Fig. 4, all MPs rescued the high tillering phenotype of the *d10* mutant, similar to GR24. The recorded number of tillers of *d10* was four tillers per plant on average in untreated (Mock) plants; this was reduced to one tiller per plant in all MP- and GR24-treated samples (Fig. 4A). In contrast, neither MPs nor GR24 could restore the high-tillering phenotype of the SL-insensitive *d3* mutant that maintained an average of four tillers per plant (overall average of all tested compounds) in treated and untreated samples (Fig. 4A, B). This result suggests that MPs act through the SL signaling pathway that requires the D3 protein. We determined the EC_50_ values of MP1, MP3, and MP7 for reducing the high-tillering phenotype of the *d10* mutant, using concentrations ranging from 2.5 × 10^–6^ to 2.5 × 10^–12^ M and in comparison to GR24. MP3 showed the lowest EC_50_ value (2.98 × 10^–9^ M), followed by MP7 (2.54 × 10^–8^ M), GR24 (4.83 × 10^–2^ M) and MP1 (2.42 × 10^–1^ M) (Fig. 4C). This result suggests that MP3 and MP7 are more active than GR24 in inhibiting rice tillering, with MP3 being the most active compound (Fig. 4C and Supplementary Fig. S4). Besides reducing the number of tillers, we observed that treatment with MP2, MP3, MP6, MP9, and MP10 at 2.5 μM, concentration stunted the growth and caused senescence symptoms of rice seedlings, leading to an obvious decrease in biomass (Supplementary Fig. S3). We assumed that this effect is caused by the high activity of these compounds in inducing senescence.

### MP3 accelerates dark-induced leaf senescence

**Fig. 5.**
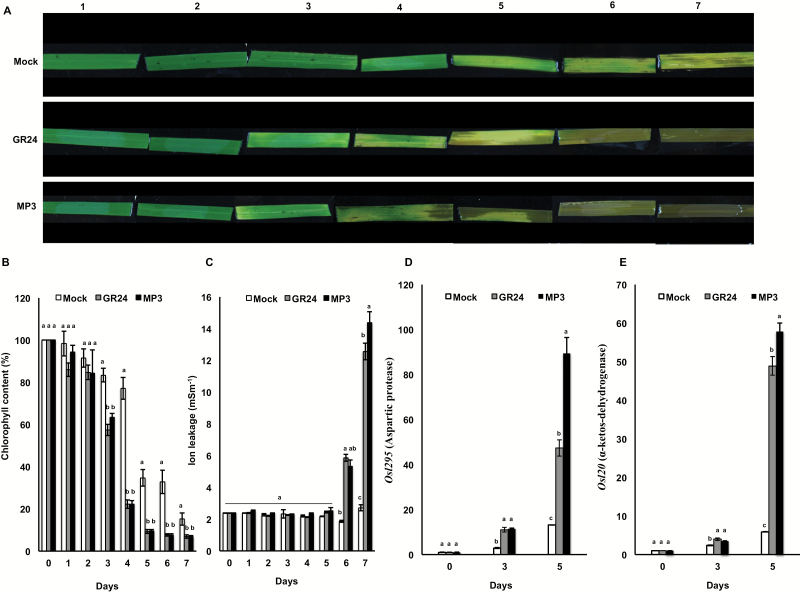
Measurement of dark-induced leaf senescence, chlorophyll content, ion leakage and transcript level of senescence-associated genes (SAGs) in response to MP3 and GR24. (A) Changes in rice leaf color in response to MP3 and GR24. Treated leaf segments were monitored over a 7 d period. (B) Chlorophyll content, (C) membrane ion leakage, (D, E) transcript level of SAGs (*OsI20* and *OsI295*) in the leaf segments was measured on the 1st, 3rd and 5th day after application. Data are means±SE (*n*=3). Means not sharing a letter in common differ significantly at *P*_0.05_.

We also investigated the effect of MP3 on dark-induced leaf senescence, in comparison with GR24. We observed a change in color in GR24- and MP3-treated leaf segments already on the third day after treatment and about 2 d earlier than the control (Fig. 5A). Consistently, measurement of the chlorophyll content showed a clear reduction in GR24- and MP3-treated segments on the third day, which further increased in the following days (Fig. 5B). We also measured the ion leakage that is usually caused by senescence. As shown in Fig. 5C, we did not detect a difference between GR24- or MP3-treated segments and the control in the first 5 d. However, on the sixth and seventh day, both GR24- and MP3-treated leaf segments showed a striking increase, compared with the control. Finally, we determined the transcript levels of the senescence-associated genes (SAGs) *Osl20* (coding for branched chain α-keto dehydrogenase) and *Osl295* (coding for aspartic protease) (Lee *et al.* 2001). After 5 d of treatment, both genes showed a striking increase in their transcripts, which was more pronounced in MP3-treated samples (Fig. 5D, E).

### Effect of MPs on seedlings development in Arabidopsis

**Fig. 6.**
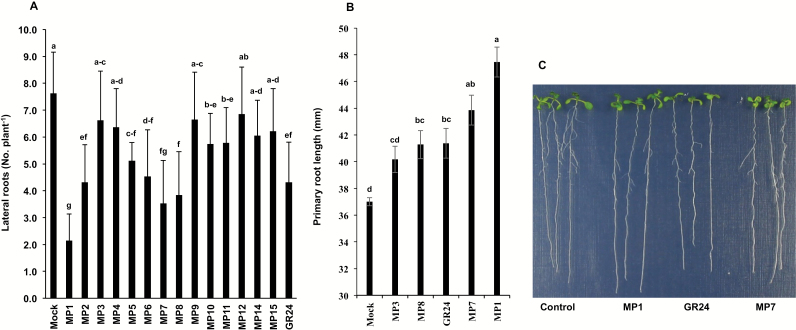
Effect of MPs on Arabidopsis lateral root density and primary root length. (A) Number of lateral roots per plant after MP application. Eight-day-old seedlings (at least 30) treated with selected MPs were photographed digitally, and then measurement was carried out using ImageJ software. Bars represent means+SE. (B) Primary root length in response to the application of MP1, MP3, MP7, MP8, and GR24. Bars represent means±SE. Means not sharing a letter in common differ significantly at *P*_0.05_. (C) A representative image showing effect of MP1, MP7, and GR24 on roots architecture.

We also tested the effect of MPs on hypocotyl elongation in Arabidopsis seedlings. Apart from MP12 and MP15, all MPs showed significant inhibition of hypocotyl growth, particularly MP7, which displayed a stronger inhibitory effect than GR24 that was applied at the same concentration (1.0 μM; Supplementary Fig. S5). We also investigated the effect on lateral root density. Here again, all compounds reduced the number of lateral roots with different efficiencies. MP1 was the most efficient compound and showed an activity higher than that of GR24, followed by MP7 and MP8, which were slightly more active than GR24 (Fig. 6A). We also tested the activity of MPs in increasing primary root length (Fig. 6B). In this experiment, we evaluated the lead molecule MP3 and three compounds, MP1, MP7 and MP8, that showed the highest activity in decreasing lateral root densities. MP1 was the most active compound, followed by MP7 and the positive control, GR24. MP3 showed a weaker activity than GR24 and MP8. The latter two caused a similar increase in primary root length (Fig. 6C).

### MP1 is the preferred substrate for ShHTL7 and OsD14

**Fig. 7.**
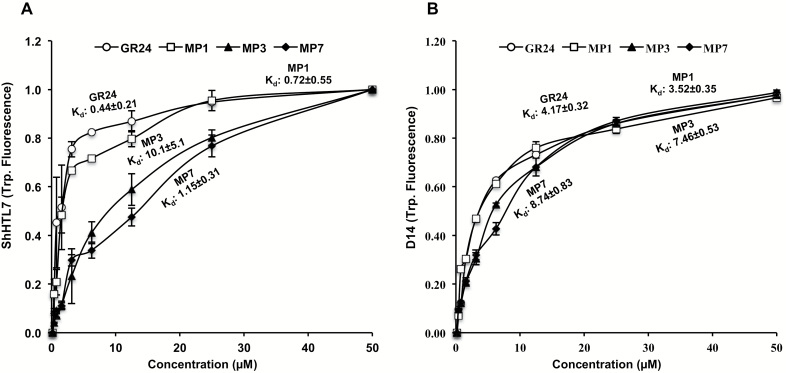
Affinity of ShHTL7 (A) and OsD14 (B) for MPs. The affinity of ShHTL7/D14 towards GR24 and selected MPs was determined using intrinsic tryptophan fluorescence assays. Changes in fluorescence were used to calculate the dissociation constant (*K*_d_). Data points represent means±SE (*n*=3).

**Fig. 8.**
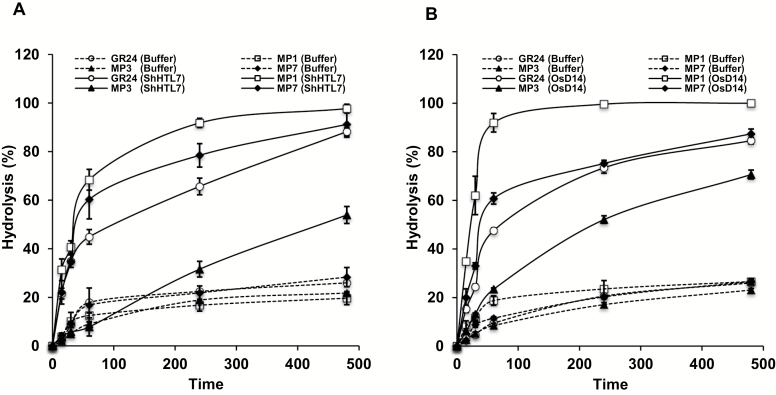
Hydrolysis of MPs by ShHTL7 and OsD14. (A) Hydrolysis of GR24 and selected MPs by ShHTL7. (B) OsD14 was monitored by HPLC at the indicated time points (with or without purified ShHTL7 or OsD14). Values represent means±SE (*n*=3).

To better understand the differences in inducing germination of *S. hermonthica* seeds, we determined the affinity of ShHTL7 for MP1, MP3, and MP7 by measuring its intrinsic tryptophan fluorescence upon binding to different concentrations of these compounds and in comparison with GR24. ShHTL7 exhibited the highest affinity towards GR24 with a *K*_d_ value of 0.44 ± 0.21 µM (Fig. 7A). We observed a slightly weaker affinity for MP1 (0.72 ± 0.55 µM), followed by MP7 (1.15 ± 0.31 µM), and the lowest affinity for MP3 (10.1 ± 5.1 µM) (Fig. 7A). To test affinity of MPs towards OsD14, we also measured intrinsic tryptophan fluorescence upon binding to different concentrations of selected MPs (1, 3 and 7) in comparison with GR24. As depicted in Fig. 7B, OsD14 showed the highest affinity towards MP1 (3.5 ± 0.35 µM), followed by GR24 (4.17 ± 0.32 µM), MP3 (7.46 ± 0.53 µM), and finally MP7 (8.74 ± 0.83 µM). We investigated the hydrolysis rates of three MPs by SHTL7, in comparison with GR24. For this purpose, we incubated purified SHTL7 with the compounds and monitored hydrolysis by HPLC after defined time intervals. As shown in Fig. 8A, we observed the highest hydrolysis rates with MP1, followed by MP7, GR24, and finally MP3. We also tested hydrolysis of three MPs by purified OsD14. Here again, we observed the same tendency, by detecting the highest hydrolysis rate with MP1, followed by MP7, GR24, and finally MP3 (Fig. 8B).

## Discussion

SLs regulate different aspects of plant development and responses to environmental changes. Moreover, they play a key role in the life cycle of root parasitic weeds and in establishing the plant beneficial mycorrhizal symbiosis. Therefore, SLs have a great potential for application in agriculture, horticulture, and forestry (Screpanti *et al.*, 2016). Sources of natural SLs are quite limited, since plants usually produce these compounds at very low concentrations (exudates of 300 000 sorghum plants were required to isolate only 5 μg of sorgolactone; Humphrey *et al.*, 2006). Moreover, organic synthesis of natural SLs is challenging, due to their complex structures that also contains several chiral centers (Zwanenburg *et al.*, 2013). Therefore, there is a need for low-cost and efficient SL analogs/mimics. Developing SL analogs with specificity for particular function(s) would also take an important step in translating SL research into potential applications.

We were particularly interested in developing SL analogs that can be applied as a suicidal seed germination agent to combat the root parasites *S. hermonthica* and *P. ramosa*. The suicidal germination approach would address a major constraint in the control of root parasitic weeds, which is the generally observed large size of seed bank of parasite-infested soil. Seasonal applications of germination stimulants in the absence of a host would gradually deplete this seed bank. This strategy has been recently tested in *P. ramosa*- and *S. hermonthica*-infested tobacco and sorghum fields, respectively (Samejima *et al.*, 2016; Zwanenburg *et al.*, 2016*a*). Results obtained from these field trials are promising and demonstrate the feasibility of the suicidal germination approach and consequently the importance of developing optimized SL analogs.

Most of our knowledge about SL functions was deduced from experiments performed with the widely used SL analog GR24. However, the synthesis of this compound is laborious and requires six to eight steps (Mangnus *et al.*, 1992*b*). Similarly, the synthesis of the potent fluorescent analog CISA-1 requires eight steps (Rasmussen *et al.*, 2013). Other compounds, such as AR36 (Boyer *et al.*, 2014) and 4-Br-debranone (4BD) (Fukui *et al.*, 2013), which contain a D-ring connected by an enol ether bridge to less complex second moieties, showed moderate activity in inducing parasite seed germination. Nijmegen-1 is a potent inducer of seed germination in *P. ramosa*, but with an EC_50_ that is three orders of magnitude higher than GR24, it causes a weak germination response of *S. hermonthica* (Wigchert *et al.*, 1999). In this study, we developed a new series of SL analogs, MPs that resemble the non-canonical SL methyl carlactonoate. MPs are easy to prepare and very potent in inducing seed germination of *S. hermonthica* seeds and in exerting several SL developmental functions, such as inhibition of tillering, triggering senescence and regulation of root architecture in Arabidopsis.

Previously, we designed a carlactone-based analog, nitro-phenlactone, that showed very weak activity in stimulating *S. hermonthica* germination (Jia *et al.*, 2016). In this work, we modified the structure of nitro-phenlactone by replacing the methyl group by a methyl caroboxy group, which led to MP1, the most efficient *Striga* germination stimulant (EC_50_=1.5 × 10^–9^ M) among the MPs tested here. This activity was around 17 times weaker than that of GR24, pointing to MP1 as a suitable candidate for the suicidal germination approach. Very recently, we have evaluated the activity of MP1 in a trial conducted in Burkina Faso, in which we used 1 × 1 m^2^ wooden boxes filled with artificially infested soil and pearl millet as a host. In this experiment MP1 showed significantly higher germination activity than GR24 (to be published). The activity of MP1 in inducing seed germination in *P. ramosa* was quite similar to that of GR24 and only slightly weaker than that of MP2, which carries the nitro group at the *ortho* position, and of the chlorophenyl compound MP8. Apart from these three compounds, we observed lower germination activity with *P. ramosa* seeds than with those of *S. hermonthica*.

To test whether MPs can recover the high-tillering phenotype of the SL-deficient *d10*/*CCD8* mutant, we used a hydroponic culture system (Fig. 4). The tillering inhibition observed with all MPs in the *d10* mutant and insensitivity of the high-tillering perception mutant *d3* prove that these compounds act as SL analogs via the F-box protein D3/MAX2-dependent signaling pathway. The high activity of MP3 and MP7 prompted us to quantify the effect on tillering in more detail and to determine the corresponding EC_50_ value. The results obtained demonstrate that both MP3 (EC_50_=2.98 × 10^–9^ M) and MP7 (EC_50_=2.54 × 10^–8^ M) are more efficient than GR24 (EC_50_=4.83 × 10^–2^ M) in restoring wild type tillering to the *d10* mutant. MP1, which showed the highest activity in the *S. hermonthica* seed germination test, was weaker than GR24. We also observed that the treatment with several MPs (at 2.5 μM), particularly MP3, leads to growth retardation and senescence. Therefore, we tested the activity of MP3 in accelerating dark-induced leaf senescence, as reported for SLs before (Yamada *et al.*, 2014). In this experiment, treatment with MP3 accelerated the change in leaf color and led to a decrease in chlorophyll content and an increase in electrolyte leakage, similar to GR24. Supporting data were also obtained by investigating the changes in transcript levels of the senescence-associated genes *Osl295* and *Osl20*. These results suggest the possibility of developing MP-based post-emergence herbicides.

The growth-promoting effect of widely used GR24 on primary roots is subtle and depends on the plant species and growth conditions (Ruyter-Spira *et al.*, 2013). Moreover, it is less pronounced than the inhibitory effect of this compound on lateral root densities (Matthys *et al.*, 2016). Our study on the activity of MPs on root architecture in Arabidopsis demonstrates that MP7 and particularly MP1 are more efficient than GR24 in inducing the growth of primary roots and reducing the lateral root density. These results suggest that MP1 and, to a lesser extent, MP7 are better analogs for investigating the role of SLs in regulating root architecture in Arabidopsis and, likely, in other species. Interestingly, nitro-phenlactone, which lacks the enone moiety, did not affect lateral root density as reported by Jia *et al.* (2016). To our knowledge, MP1 is the first reported analog that outperforms GR24 in impacting root development.

Comparison of the activities of different MPs unraveled the impact of substitutions in the phenyl ring on the efficiency of these compounds in exerting specific SL functions. The lead compound, MP3, was less active than MP1 in inducing *S. hermonthica* seed germination but showed the most pronounced inhibitory effect on tillering. Substitutions increased the efficiency of the compounds in inducing seed germination and in regulating root architecture. Besides being the most potent compound in the *S. hermonthica* seed germination assay, MP1 also showed the highest activities in repressing the number of lateral roots and enhancing the length of primary roots, followed by the chlorophenyl-containing MP7 and MP8. The position of the modification is decisive. Among the chlorophenyl compounds, MP7, MP8, and MP9, we observed highest germination activity with MP7 (Cl in *para* position) followed by MP9 (*ortho* position) and MP8 (*meta* position).

To shed light on molecular events underlying the differences in activity between MP1, MP3, and MP7, we determined the affinity of *S. hermonthica* SL receptor ShHTL7 for these compounds. The results obtained were consistent with the *S. hermonthica* germination assay, with GR24 being the most active ligand followed by slightly weaker MP1, MP7, and finally MP3. However, we observed higher hydrolysis activities with MP3 and MP7 than with GR24. We also compared these compounds regarding their affinity and hydrolysis by the rice SL receptor D14. MP1 exhibited the highest hydrolysis rate and the lowest *K*_d_, followed by GR24, MP3, and finally MP7. It may be speculated that the high conversion of MP1 and MP7 is due to the presence of electron withdrawing groups NO_2_ (MP1) and Cl (MP7), which alleviate the hydrolysis by the two receptors. In the case of ShHTL7 assays, hydrolysis and affinity results are consistent with the determined biological activity. In contrast, MP3 was the less preferred substrate in the incubations with OsD14, although it showed the highest activity in the tillering assay. This difference indicates that other factors, such as uptake and transport, are also decisive for the tillering inhibitory and growth retarding activity. However, it is also possible that hydrolysis rates determined here do not accurately reflect the situation *in planta* where D14 is part of a protein complex that may impact or modulate hydrolysis activity of this receptor.

## Conclusions

Our findings showed that MPs are highly efficient SL analogs that can be used to investigate the biological functions of SLs and employed to combat root parasitic weeds or to modulate plant architecture. Moreover, our study demonstrated that type and position of substitutions in the phenol ring, which corresponds to the A-ring in SLs, determine and modulate the efficiency of MPs in exerting specific SL functions.

## Supplementary data

Supplementary data are available at *JXB* online.

Fig. S1. Stability analysis of MPs in comparison with GR24.

Fig. S2. Parasitic seed germination in response to MP treatment.

Fig. S3. Effect of MPs on rice fresh biomass. 

Fig. S4. Images showing the effect of selected MPs (MP1, MP3, MP7) and GR24, applied at concentration ranging from 2.5 μM to 2.5 × 10^−7^ μM, on tillering and growth of *d10* seedlings.

Fig. S5. Effect of MPs on Arabidopsis hypocotyl length. 

Table S1. Methyl phenlactonoates: physico-chemical properties.

Table S2. List of primer sequences used in the study.

Table S3. Yield and quantity of synthesized MPs.

erx438_suppl_supplementary_figures_s1_s5_tables_s1_s3

## Data deposition

The supplementary tables and figures are also available at the Dryad Data Repository: htpp://dx.doi.org/10.5061/dryad.hq7b8

## Author contributions

MJ designed experiments, performed *Striga* and tillering bioassays, studied leaf senescence, and wrote the manuscript. BAK helped in parasitic seed and tillering bioasays, data compiling and analysis. IH, STA, and USH conducted ShHTL7/OsD14 binding, hydrolysis studies. XG did stability analysis. VON conducted leaf senescence and SAG gene expression studies. KJ studied Arabidopsis hypocotyl length and lateral root inhibition. SA studied Arabidopsis primary root length. YL, JK, KH, and MT prepared and purified protein and carried out the hydrolysis. TA synthesized MPs and SAB designed and proposed the MP study.
