# Supplementary material for: Methyl phenlactonoates are efficient strigolactone analogs with simple structure
Source: J Exp Bot. 2017 Dec 28;69(9):2319–31. doi: 10.1093/jxb/erx438 (PMC5913645; doi:10.1093/jxb/erx438)
Supplement: erx438_suppl_supplementary_figures_s1_s5_tables_s1_s3 [file erx438_suppl_supplementary_figures_s1_s5_tables_s1_s3.pdf]

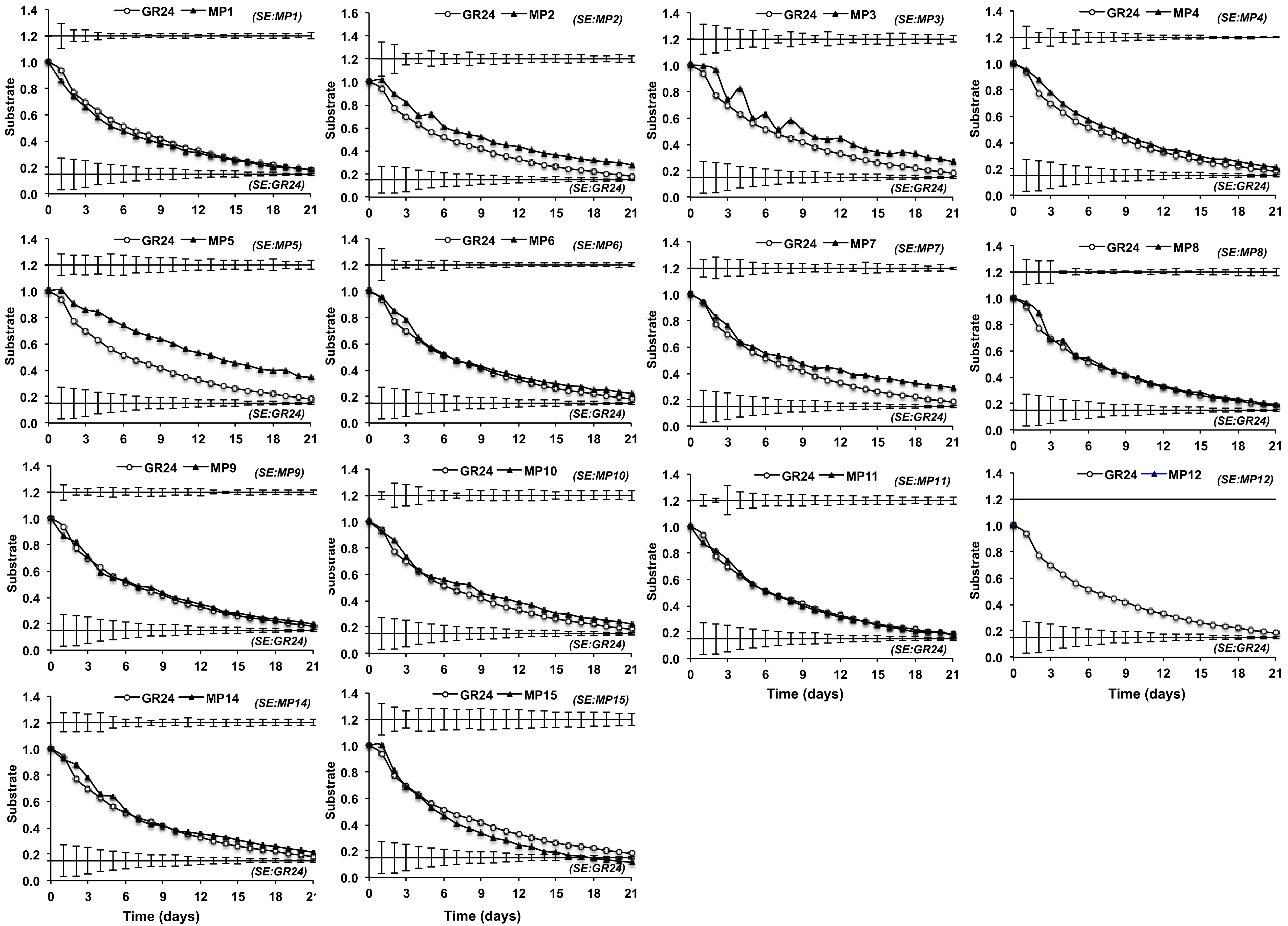

Fig. S1. Stability analysis of MPs in comparison to GR24. The stability of MPs was monitored daily for 3 weeks. Data are means ± SE (n=3). X-axis (time (days)); Y-axis (substrate).

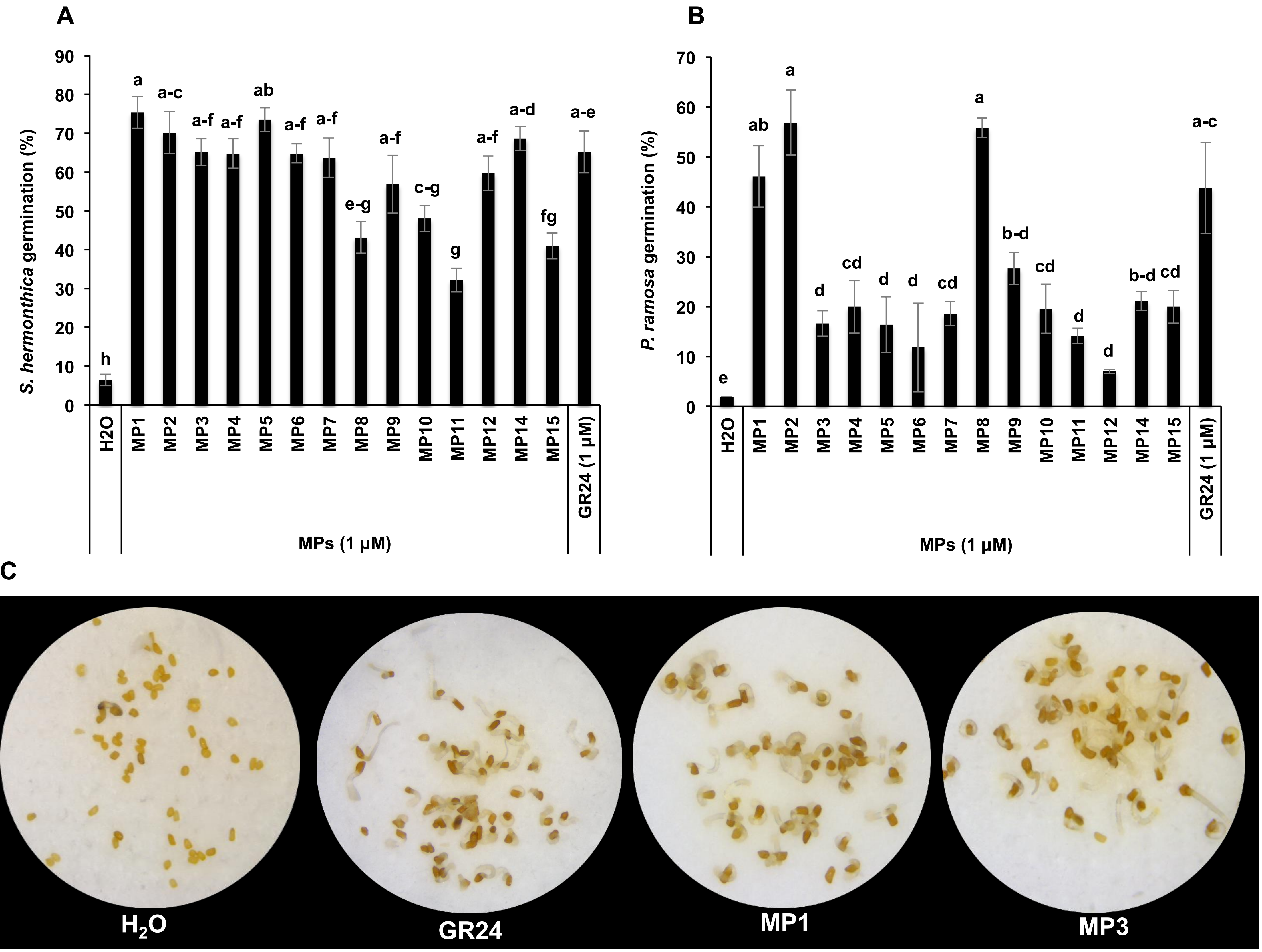

Fig. S2. Parasitic seed germination in response to MPs treatment. (A) Each MP was applied in 50 µl volume (1.0 µM) on a disc containing 50-100 pre-conditioned *Striga* seeds. H2O and GR24 are included as negative and positive control, respectively. (B) *P. ramosa* seed germination in response to MPs application. Each MP (1.0 µM) was applied in 50 µl volume on a disc containing 50-100 preconditioned *P. ramosa* seeds. Bars represented means ± SE (*n*=3). Means not sharing a letter in common are differ significantly at $P_{0.05}$. (C) Picture showing response of pre-conditioned *S. hermonthica* seed germination for H_2O, GR24, MP1 and MP3 application.

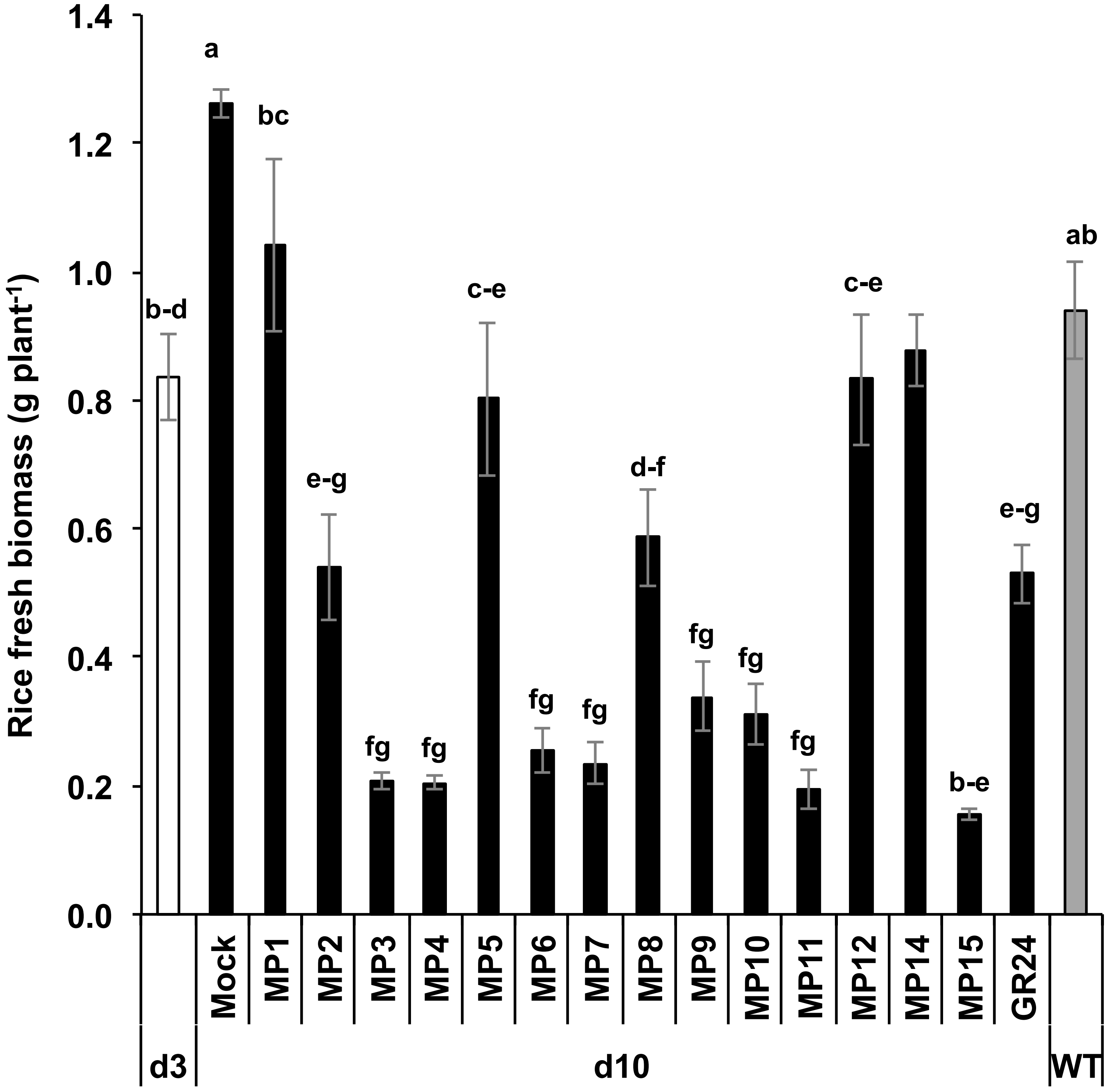

Fig. S3. Effect of MPs on rice fresh biomass. MPs were applied (2.5 µM) to one week old hydroponically grown rice seedlings (Shiokari, *d3*, *d10*) twice a week for three weeks. Bars represent means ± SE (*n*=8). Means not sharing a letter in common are differ significantly at $P_{0.05}$.

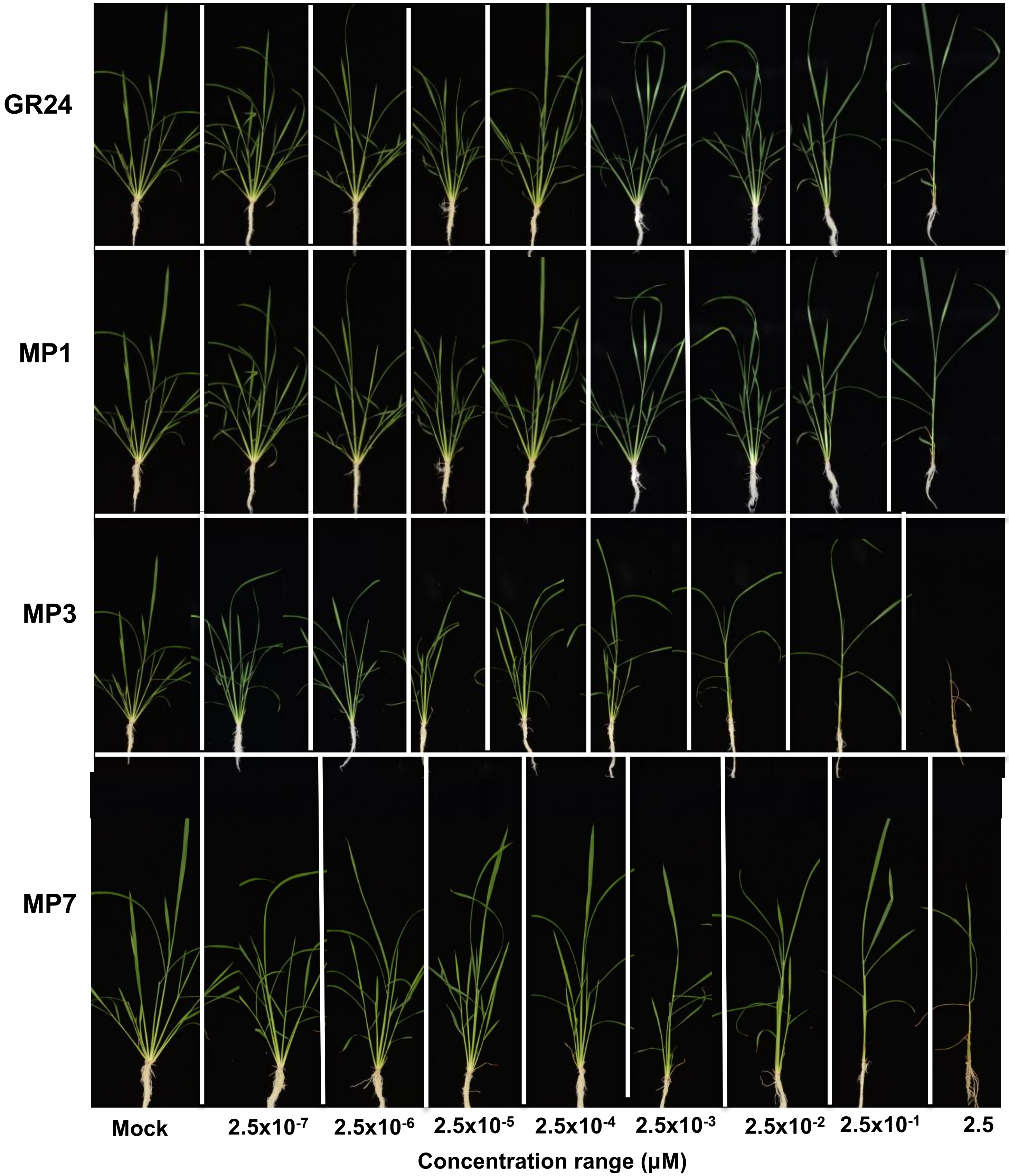

Fig. S4. Picture showing the effect of selected MPs (MP1, MP3, MP7) and GR24, applied at concentration ranging from 2.5 μM-2.5x10^{-7} μM, on tillering and growth of *d10* seedlings.

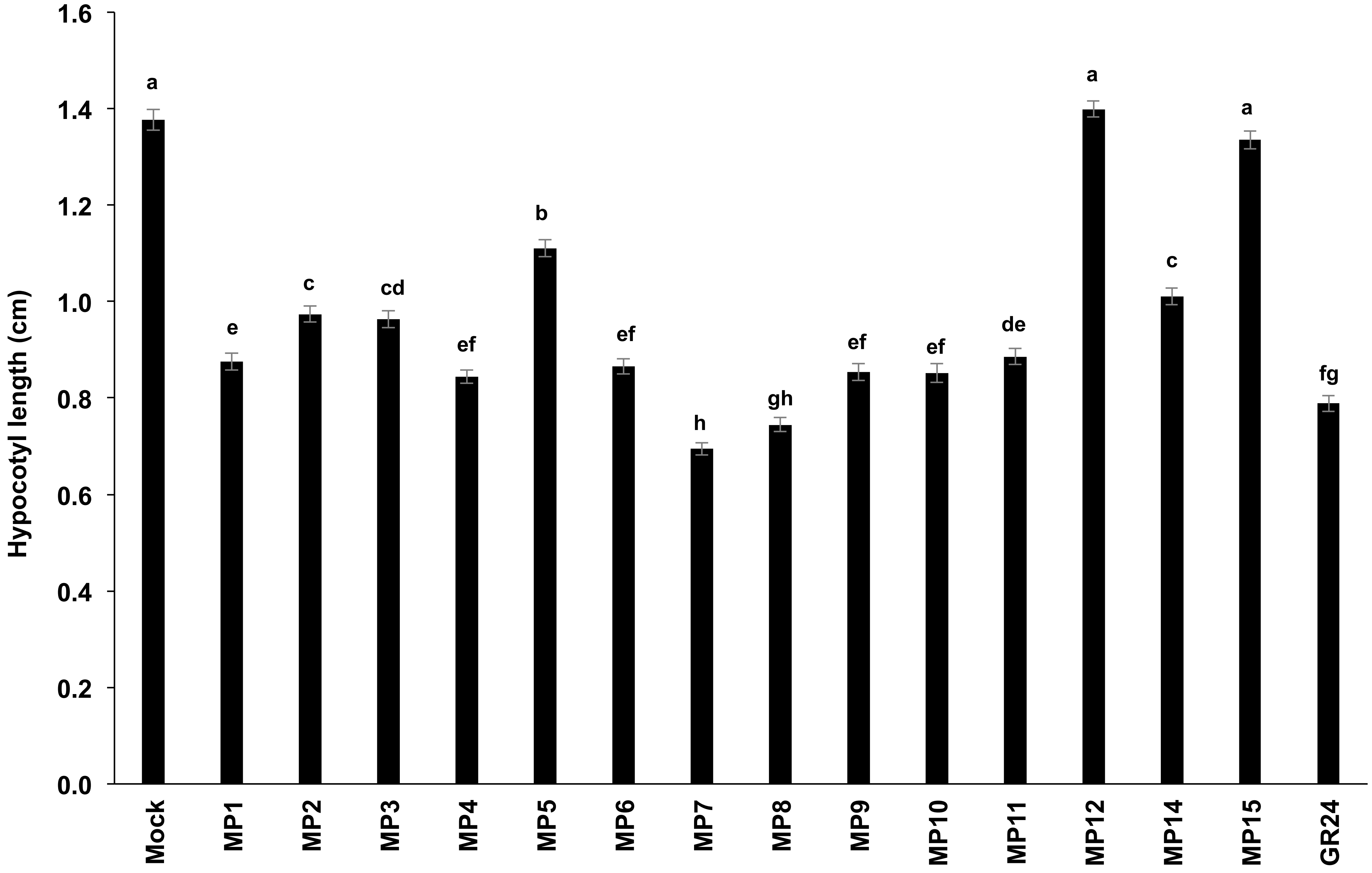

Fig. S5. Effect of MPs on Arabidopsis hypocotyl length. Eight days old Arabidopsis seedlings (at least 30) treated with selected MPs were photographed digitally and, measurement of the hypocotyl length was conducted using ImageJ software. Bars represent means ± SE. Means not sharing a letter in common are differ significantly at $P_{0.05}$.

Table S1. Methyl Phenlactonoates: Physico-Chemical Properties

Z/E stereochemistry was assigned based on NOE experiments. In case of E-isomer NOE between vinyl proton and aromatic proton was observed. In case of Z-isomer NOE between vinyl proton and aromatic proton was not observed and instead NOE between vinyl proton and methyl proton of carbomethoxy group was observed.

**MP1**	^{1}H NMR (500MHz, $CDCl_3$): d8.20 (2H, d, *J* = 9 Hz), 7.84 (1H, s), 7.49 (2H, d, *J* = 8.5 Hz), 6.87 (1H, s), 6.17 (1H, s), 3.79 (3H, s), 2.00 (3H, s); ^{13}C NMR (125MHz, CDCl) δppm 170.29, 166.23, 154.38, 147.01, 141.02, 138.69, 135.89, 132.22, 123.08, 113.82, 100.67, 52.26, 10.76; IR (nujol mull method): v_{max} 1780, 1710, 1670, 1505, 1350 cm^{-1}. HRMS-ESI: m/z $[M-Na]^-$ Calcd for $C_{15}H_{13}N_1Na_1O_7$:342.05897, found: 342.05741. Yield: 9%.
**MP2**	^{1}H NMR (500MHz, $CDCl_3$): d8.05 (1H, d, *J* = 8 Hz), 7.75 (1H, s), 7.60 (1H, t, *J* = 7.5 Hz), 7.48 (1H, t, *J* = 8 Hz), 7.37 (1H, d, *J* = 7.5 Hz), 6.83 (1H, s), 6.13 (1H, s), 3.72 (3H, s), 1.97 (3H, s). HRMS-Esi: m/z $[M-Na]^-$ Calcd for $C_{15}H_{13}N_1Na_1O_7$:342.05897, found: 342.05784. Yield: 17%.
**MP3**	^{1}H NMR (500MHz, $CDCl_3$): d7.74 (1H, s), 7.34 (2H, d, *J* = 7.5 Hz), 7.30 (3H, m,), 6.85 (1H, s), 6.14 (1H, s), 3.76 (3H, s), 1.97 (3H, s); ^{13}C NMR (125MHz, CDCl) δppm 170.41, 167.18, 152.61, 141.26, 135.33, 131.51, 129.97, 127.77, 127.52, 115.54, 100.40, 51.83, 10.55; IR (nujol mull method): v_{max} 1790, 1770, 1670 cm^{-1}. HRMS-ESI: m/z $[M-Na]^-$ Calcd for $C_{15}H_{14}Na_1O_5$:297.07389, found: 297.07252. Yield: 50%.
**MP4**	^{1}H NMR (500MHz, $CDCl_3$): δ7.71(1H, s), 7.18 (4H, m), 6.85 (1H, s), 6.13 (1H, s), 3.76 (3H, s), 2.35 (3H, s), 1.97 (3H, s). HRMS-ESI: m/z $[M-Na]^-$ Calcd for $C_{16}H_{16}Na_1O_5$:311.0895, found: 311.08907. Yield: 48%.
**MP5**	^{1}H NMR (500MHz, $CDCl_3$): δ7.90 (1H, s), 7.81 (1H, d, *J* = 7.5 Hz), 7.34 (1H, t, *J* = 8 Hz), 7.27 (1H, t, *J* = 8 Hz), 7.17 (1H, d, *J* = 7.5 Hz), 7.03 (1H, s), 6.29 (1H, s), 5.29 (2H, dd, *J* = 18.5, 8.5 Hz), 2.05 (3H, s). HRMS-ESI: m/z $[M-Na]^-$ Calcd for $C_{15}H_{12}Na_1O_5$:295.05824, found: 295.05756. Yield: 20%.
**MP6**	^{1}H NMR (500MHz, $CDCl_3$): δ7.75(1H,s), 7.30(1H, q, *J* = 7 Hz), 7.08 (1H, d, *J* = 6.5 Hz), 7.01 (2H, m), 6.87 (1H, s), 6.14 (1H, s) 3.77 (3H, s), 1.99 (3H, s). HRMS-ESI: m/z $[M-Na]^-$ Calcd for $C_{15}H_{13}F_1Na_1O_5$:315.06447, found: 315.06407. Yield: 59%.
**MP7**	^{1}H NMR (500MHz, $CDCl_3$): δ7.74(1H,s), 7.31 (2H, d, *J* = 8.5 Hz), 7.24 (2H, d, *J* = 8.5 Hz), 6.86 (1H, s), 6.13 (1H, s), 3.77 (3H, s), 1.99 (3H, s). HRMS-ESI: m/z $[M-Na]^-$ Calcd for $C_{15}H_{13}Cl_1Na_1O_5$:33103492, found: 331.03461. Yield: 60%.

**MP8**	^1H NMR (500MHz, $CDCl_3$): δ7.77(1H,s), 7.30 (3H, m), 7.20 (1H, m), 6.89 (1H, s), 6.16 (1H, s), 3.79 (3H, s), 2.01 (3H, s). HRMS-ESI: m/z $[M\text{-}Na]^-$ Calcd for $C_{15}H_{13}Cl_1Na_1O_5$:33103492, found: 331.03503. Yield: 70%.
**MP9**	^1H NMR (500MHz, $CDCl_3$): δ7.75(1H,s), 7.41 (1H, m), 7.26 (2H, m), 7.21 (1H, m), 6.84 (1H, s), 6.13 (1H, s), 3.74 (3H, s), 1.96 (3H, s). HRMS-ESI: m/z $[M\text{-}Na]^-$ Calcd for $C_{15}H_{13}Cl_1Na_1O_5$:33103492, found: 331.03401. Yield: 60%.
**MP10**	^1H NMR (500MHz, $CDCl_3$): δ7.39(1H,d, *J*= 7 Hz), 7.28 (3H, m), 7.06 (1H, s), 6.82 (1H, s), 6.16 (1H, s), 3.71 (3H, s), 2.01 (3H, s). HRMS-ESI: m/z $[M\text{-}Na]^-$ Calcd for $C_{15}H_{13}Cl_1Na_1O_5$:33103492, found: 331.03452. Yield: 4%.
**MP11**	^1H NMR (500MHz, $CDCl_3$): (Mixture of *Z*- and *E*- stereoisomers. *Z*:*E* = 6.7:1) δ7.96 (1H, s, *Z* isomer), 7.79 - 7.88 (2H for *Z* isomer and 3H for *E* isomer, m), 7.69 (1H, m, *Z* isomer), 7.43 - 7.51 (3H for *Z* isomer and 3H for *E* isomer, m), 7.37 (1H, d, *J* = 7 Hz, *E* isomer,), 7.32 (1H, d, *J* = 7 Hz, *Z* isomer), 7.17 (1H, s, *E* isomer), 6.89 (1H, s, *E* isomer), 6.70 (1H, s, *Z* isomer), 6.16 (1H, s, *E* isomer), 6.11 (1H, s, *Z* isomer), 3.70 (3H, s, *Z* isomer), 3.64 (3H, s, *E* isomer), 2.01(3H, s, *E* isomer), 1.89(3H, s, *Z* isomer). HRMS-ESI: m/z $[M\text{-}Na]^-$ Calcd for $C_{19}H_{16}Na_1O_5$:347.08954, found: 347.08909. Yield: 38%.
**MP12**	^1H NMR (500MHz, $CDCl_3$): δ9.15(1H,s), 8.23 (2H, d, *J* = 9 Hz), 7.60 (2H, d, *J* = 8.5 Hz), 7.44 (1H, s), 6.97 (1H, s), 6.29 (1H, s), 2.04 (3H, s). HRMS was not obtained. Yield: 27%.
**MP14**	^1H NMR (500MHz, $CDCl_3$): (Mixture of *Z*- and *E*- stereoisomers. *Z*:*E* =7.7:1) δ7.70 (1H, s, *Z* isomer), 7.24 (2H d, *J* = 8.5 Hz, *Z* isomer) 7.21 (2H d, *J* = 8.5Hz, *E* isomer), 7.03 (1H, s, *E* isomer), 6.90 – 6.86 (3H for *Z* isomer and 2H for *E* isomer, m), 6.82 (1H, s, *E* isomer), 6.13 (1H, s, *Z* isomer), 6.12(1H, s, *E* isomer), 3.81 (3H, s, *E* isomer), 3.80 (3H, s, *Z* isomer), 3.77 (3H, s, *E* isomer), 3.76 (3H, s, *Z* isomer), 2.00 (3H, s, *E* isomer), 1.98 (3H, s, *Z* isomer). HRMS-ESI: m/z $[M\text{-}Na]^-$ Calcd for $C_{16}H_{16}Na_1O_6$:327.08446, found: 327.08362. Yield: 42%.
**MP15**	^1H NMR (500MHz, $CDCl_3$): δ7.71 (1H,s), 7.19 (2H, d, *J* = 8.5 Hz), 6.87 (1H, s), 6.81 (2H, d, *J* = 9 Hz), 6.14 (1H, s), 5.04 (1H, bs), 3.77 (3H, s), 1.99 (3H, s). HRMS-ESI: m/z $[M\text{-}Na]^-$ Calcd for $C_{15}H_{14}Na_1O_6$:313.06881, found: 313.06760. Yield: 55%.

Table S2. List of primers sequences used in this study

Primer name	Sequences (5'-3')	Restriction site	Purpose
ShHTL7-F	CGggatccATGAGCTCAATTGGATTAGCCC	*Bam*HI	Protein cloning
ShHTL7-R	CCGctcgagTCAGTGATCCGTGATGTCCTG	*Xho*I	Protein cloning
Osl20-F	TCAGGAACAATGTGAGGCAAGAG		qRTPCR
Osl20-R	CGCGGTTTCGGCATTCTTTCTG		qRTPCR
Osl295-F	TGATGGGAAGCATGGTGTTAGTG		qRTPCR
Osl295-R	CACTTTGGAGACCATTCGATTCC		qRTPCR
Ubi-F	GCCCAAGAAGAAGATCAAGAAC		qRTPCR
Ubi-R	AGATAACAACGGAAGCATAAAAGTC		qRTPCR

Table S3. Yield and quantity of synthesized MPs

MPs#	Yield (%)	Quantity obtained (mg)
MP1	9	52.1
MP2	17	55.3
MP3	50	61.7
MP4	48	44.6
MP5	20	49.4
MP6	59	53.9
MP7	60	52.6
MP8	70	47.4
MP9	60	51.6
MP10	4	26.2
MP11	38	57.3
MP12	27	53.4
MP14	42	55.2
MP15	55	46.7
